# A case of allergy to Silybum marianum (*milk thistle*) and Eragrostis tef (*teff*)

**DOI:** 10.1186/s13223-020-00421-5

**Published:** 2020-04-15

**Authors:** O. Wojas, E. Krzych-Fałta, P. Samel-Kowalik, M. Żalikowska-Gardocka, E. Majsiak, A. Mari, B. Samoliński

**Affiliations:** 1 Deprtament of Environmental Hazard Prevention and Allergology, Medical University of Warsaw, Warsaw, Poland; 2 Department of Gastroenterology and Internal Medicine, Medical University of Warsaw, Warsaw, Poland; 3 Polish-Ukrainian Foundation for the Development of Medicine, Lublin, Poland; 4 Clinician and Scientist in Allergy and Immunology Centri Associati di Allergologia Molecolare (CAAM), Rome, Italy

**Keywords:** Allergen, Allergies, Milk thistle, Silybum marianum, Teff, Eragrostis tef

## Abstract

**Background:**

This paper presents a peculiar first case of an allergy to Silybum marianum (milk thistle) and Eragrostis tef (teff). Both teff and milk thistle have been presented in the literature (both domestic and foreign) in a positive light, the former as a new part of gluten-free diet, and the latter as a treatment for a number of conditions, particularly those of the liver.

**Case presentation:**

A 29-year-old male presented at our clinic due to an episode of itching and burning in his mouth, swollen tongue, and difficulty swallowing following ingestion of teff flakes. He also reported sneezing, runny nose, watering and burning eyes, and wheezing following inhalation exposure to ground milk thistle. The patient’s occupation is associated with exposure to these allergens. The patient underwent comprehensive allergy diagnostic assessments (including skin-prick testing, serum specific IgE levels, Faber test, spirometry, and acoustic rhinometry) and gastroenterological assessments. The diagnosis was established on skin tests with native allergens (milk thistle 16/35, teff flour 22/60, negative control 0/0, histamine 3/5) provided by the patient. There are no commercially available (standardized) tests for milk thistle or teff either in Poland or anywhere else in the world.

**Conclusions:**

Milk thistle is available in the form of dry, finely-ground preparations (which are used as additives to bread, soups, and yoghurts) and extracts (which are used as ingredients in over-the-counter herbal remedies). Teff is a gluten-free cereal whose grains are rich in methionine, calcium, iron, folic acid, and antioxidants. This case report presents milk thistle and teff as potentially new allergens. A literature review revealed no similar allergy cases in Poland or elsewhere in the world.

## Background

Teff (also known as, tef, teff grass, Abyssinian love grass, Williams’ love grass, or Eragrostis tef) is a cereal plant of the Poaceae family, occurring naturally in North-East Africa. The etymology of ‘teff’ has not been definitively established; the word may be derived from the Semitic word *thaf*, which refers to harvesting wild cereal plants [1]. According to another theory, the word teff derives from the Amharic word *teffa*, which means “lost”, the association likely due to the very small size of the plant’s grains [2]. The genus Eragrostis (“love grasses”) comprises over 350 species of wild plants that grow mainly in Africa, but also in North and South America, Asia, and Australia. Teff is a species endemic to Ethiopia and is the only cereal of the genus Eragrostis that is cultivated as a crop. The exact date when this plant was first used as a crop is unknown. However, teff had been already cultivated in the ancient times, at least 4500 years ago. Teff is an annual self-pollinated allotetraploid cereal plant [1, 2]. The seeds are small, light-weight, bare, and propagated by the wind. The shoots grow up to approximately 70 cm in height. The inflorescence has the form of spikelets, with lemmas that are rough on their outer surface. Teff can withstand poor soil and is highly resistant to harsh atmospheric conditions, including moderate droughts [3]. Teff grains are oval, with a diameter of 0.9–1.7 mm and length of 0.7–1.0 mm. Their color ranges from milky-white to dark brown. For marketing reasons teff is categorized by grain color: white (nech), red (quey), and mixed (sergegna) [2]. One important advantage of teff grains is that they can be stored for a long time while typically remaining unaffected by insects, rodents, and other pests. For this reason, teff stores in Ethiopia can be a valuable food reserve for times of famine. In Ethiopia teff has been cultivated as a crop, with the yield of 4.2 million tons in 2015 [4]. Apart from Ethiopia, teff is currently cultivated in the US, India, Australia, Bolivia, and Europe. Teff is a gluten-free cereal, whose grains are rich in methionine, calcium, iron, folic acid, and antioxidants.

Milk thistle (Silybummarianum, Cardusmarianus) is an annual plant of the Asteroideae (or Tubuliflorae) subfamily of the Asteraceae. This species has characteristic leaves that are marked with irregular, white, marble-like pattern. According to one legend these white markings came from drops of Virgin Mary’s milk that fell onto the plant when she was nursing Baby Jesus. Virgin Mary’s name may have also been the basis for the species name ‘marianum’. The fruits of plants of the Asteroideae subfamily are called cypselae. The cypsela is a dry, non-rupturing fruit, whose pericarp encloses a single seed. Since cypselae are involved in seed propagation, they are sometimes referred to as ‘seeds’, although this term is botanically inaccurate [5–8]. The medicinal use of milk thistle dates back to antiquity. The plant was known and used for remedies in ancient Egypt. The earliest references to milk thistle can be found in the writings of Theophrastus of Eresos (370–287 B.C.), Pedanius Dioscorides (40–90 B.C.), and Pliny the Elder (23–79 A.D.). These writings described the beneficial effects of milk thistle extracts on the human body. Pliny the Elder described the use of the plant’s juice and fruits as an antidote to snakebite and for “carrying off bile”. In the 16th century, the Swiss physiologist Albrecht von Haller emphasized the beneficial effects of milk thistle in treating liver diseases [5, 6].

The fruit (cypselae) of milk thistle contain silymar in, flavonoids, phytosterols, tannins, proteins and bio-amines, organic acids, vitamins C and K, sugars, mucilage, and mineral salts. The most important pharmacologically active constituent of milk thistle is silymarin, which constitutes 1.5–3% of plant matter. Silymar in is a complex mixture of flavonolignans including silybin, isosilybin, silychristin, silydianin, dehydrosilybin, silymonin, and silyhermin. Approximately 98% of flavonolignans can be found in the pericarp and test a of the cypselae. Silymar in has poor bioavailability when administered orally. This is due to the compound’s low water-solubility and the resulting ineffective intestinal absorption. However, this limitation has been overcome by the introduction of easily absorbable silybin-phosphatidylcholine complexes. Moreover, new silybin glycoconjugates (e.g. its conjugates with glucose, mannose, or galactose) combine the properties of high water solubility and potent antioxidant effects. In addition, experiments on animal models and studies in humans have demonstrated silymarin to be exceedingly safe when used in therapy. The most common side effects reported in long-term use at high doses are headaches and pruritus. Notably, no life-threatening side effects have been reported [5]. The most potent biological effects of silymarin are attributed to silybin (silibinin), which occurs in two diastereoisomers: A and B [5–7]. Long-term studies on pharmacologically active compounds derived from milk thistle have demonstrated the plant’s protective and detoxifying properties with respect to hepatic cells, as well as its anti-oxidant, anti-inflammatory, and anti-oncogenic properties. Milk thistle has found use in the treatment of liver and kidney diseases, diabetes mellitus, and cancer; the plant also has beneficial effects on lactation. In Poland (as well as elsewhere in the world) milk thistle is available in the form of dry, finely-ground preparations (which are used as additives to bread, soups, and yoghurts) and extracts (which are used as ingredients in over-the-counter herbal remedies) [9].

## Case presentation

A 29-year-old male presented at the Allergy Clinic (Allergy and Clinical Immunology Unit, Public Central Teaching Hospital in Warsaw) due to an episode of itching and burning in the mouth, tongue swelling, difficulty swallowing, feeling of anxiety, and rapid pulse, all of which developed several days earlier, within 5 min after ingesting teff flakes. Since the patient had been previously provided with an emergency kit, he immediately took 30 mg prednisone and 30 mg cetirizine orally. As his general condition improved rapidly, there was no need for adrenalin administration. The patient did not report to his doctor directly following the incident. The patient had already had a history of similar symptoms in the form of burning and itching in the mouth, albeit less severe, following ingestion of gluten-free bread from teff flour or containing teff. 8 months ago, in outpatient setting we diagnosed this patient with inhalant allergy to milk thistle. Four years prior to his presentation at our clinic, the patient started working at a production facility that manufactures healthy, organic foods, including gluten-free flours, food additives, and natural dietary supplements. During the first year of his employment there, he worked at a packaging station for dry, finely-ground fruit of milk thistle. At that time, he experienced no health issues. For the last 3 years, the patient has held an executive position and had no direct exposure to the packaging premises. However, he reported about a year’s history of rhinorrhea, sneezing, burning watery eyes, and wheezing whenever he came in contact with even minute amounts of milk thistle. The patient denied ever having consumed milk thistle in the form of infusions, teas, food additives, or pharmaceutical form (tablet) excipients. Milk thistle fruit is delivered to the plant already in a dry, finely ground form, which is not further processed, but only packaged on the premises. Milk thistle is delivered from an organic food farm in a defatted, ground form. The product has an appropriate quality certificate. For approximately 1 year the facility has been importing teff cereal from the Netherlands and manufacturing gluten-free flours and flakes. The facility has a product-quality certificate, containing information on the product’s sensory properties, storage conditions, physicochemical requirements, nutritional value, and allergens. The patient has not been diagnosed with celiac disease nor is on any special diet. However, as a co-owner of the facility, he samples all the foods products manufactured on the premises. It was during such routine sampling of teff-flour bread and flakes that the patient first noticed the symptoms listed above.

*Past medical history* revealed no major health issues and no current medication. His family history was negative for allergies. The patient denied hypertension, coronary artery disease, diabetes mellitus, and peptic ulcer disease. He reported periodic burning sensation in his mouth, heartburn, and dysphagia following ingestion of certain raw fruit and vegetables (apples, pears, plums, carrots, celery root). The patient had been stung twice by a wasp and developed considerable local reaction which, however, required no medical intervention. Nonetheless, 2 years prior to presentation, a wasp sting produced chest tightness and wheezing as well as localized edema and erythema. At that time, the patient was examined at an emergency room; however, he no longer has any medical records from the incident nor remembers what kind of treatment he received.

*Physical examination* revealed no apparent abnormalities. Otorhinolaryngological examination findings were as follows: Nose—no nasal septum deviation; pink, moist mucosa, slight hypertrophy of the inferior turbinates; no polyps or other growths; Pharynx—a normal tongue, with no coating; symmetrical palatal arches; palatal tonsils present in their anatomical location, no pathological discharge; clear posterior pharyngeal wall; Ears—bilateral otoscopy revealed no abnormalities; Larynx—normal appearance and function. Auscultation revealed normal breath sounds over both lung fields, no murmurs, and a regular heartbeat. The abdomen was soft, nontender. The skin was clear, with no evidence of exanthema.

*Allergy diagnostics*. Spirometry results were normal (FEV1 117% (5.14 L); FEV1/FVC 97%, 34th percentile, SR–0.42); rhinomanometry results were within normal limits. Skin prick testing (SPT) and skin tests with native alimentary allergens (from the foods brought by the patient) revealed high reactivity to the allergens of milk thistle (16/35) and teff flour (22/60), with the negative control score of 0/0, and histamine score of 3/5. Skin tests with Allergopharma allergens, sIgE (Table 1), and Faber test (Table 2) were also conducted. In order to verify the results of skin tests with native food allergens, the same tests with teff allergens were performed in 5 healthy volunteers and 5 volunteers diagnosed with an inhalant allergy to grasses, yielding negative results. Skin tests with native milk thistle allergens were also conducted in 5 healthy volunteers, yielding negative results.

**Table 1:** Allergy diagnostic assessments: skin-prick tests, skin (prick-by-prick) tests with native allergens, and allergen-specific IgE tests

Skin-prick tests			Skin (prick-by-prick) tests with native allergens			Allergen-specific IgE	
Allergen	W	F	Allergen	W	F	Allergen	Result
Chicken egg	4	5	Teff flour	22	60	wasp (i03)	1.6 kU/L (allergen class 2)
			Milk thistle	16	35		
Hazelnut	3	4	Oat flour	5	25	Speckled alder pollen (t02)	0.72 kU/L (allergen class 2)
Rye flour	3	10	Buckwheat flour	5	15	Oak pollen (t07)	0.43 kU/L (allergen class 1)
Wheat flour	3	5	Columbus grass	17	35	Rye pollen (g12)	0.55 kU/L (allergen class 1)
Anise	3	10	Rice flour	6	25	Common buckwheat (f11)	0.39 kU/L (allergen class 1)
Banana	3	15	Barley flour	6	10	Peanut (f13)	0.43 kU/L (allergen class 1)
Hazel	5	10	Oat flour	3	5	Rice (f09)	4.0 kU/L (allergen class 3)
Alder	5	10	Corn flour	5	20	Wheat flour (f04)	0.38 kU/L (allergen class 1)
Birch	3	0	Rice flour	16	35	Sesame (f10)	0.52 kU/L (allergen class 1)
Grasses	6	20	Positive control	3	5		
Rye pollen	3	20	Negative control	0	0		
*Tyrophagus* (mites)	3	0					
*Lepidoglyphus* (mites)	3	10					
Positive control	3	5					
Negative control	0	0					

*W* wheal, *F* flare

**Table 2:** Serum IgE specific to allergenic molecules (M) and extracts (E)

Serum IgE antibodies specific to certain allergenic molecules (M) and extracts (E)	Result (FIU/mL)
All c (onion) E	0.83
All p (leek) E	1.07
All s (garlic) E	1.54
Ama Cr (amaranth) E	1.07
Ara h (peanut) E	0.96
Ara h 3 (peanut) M (11S globulin)	1.20
Cas s (chestnut) E	1.20
Cer s (carob) E	2.02
Cic a (chickpea) E	2.14
Cit r (mandarin) E	1.19
Cor a (leszczyna) E	1.43
Cuc s (cucumber) E	1.43
Dau c (carrot) E	1.67
Fag e (buckwheat) E	5.07
Foe v (sweet fennel) E	1.30
Hor v (common barley) E	3.46
Jug r (walnut) E	1.07
Lol p (perennial rye grass) E	2.02
Lol p1 (perennial rye grass) M	1.30
Lup a (white lupin) E	0.96
Mus m (house mouse) E	2.02
Mus m 4 (house mouse) M	1.54
Ory s (rice) E	8.64
Pers a (avocado) E	0.96
Phl p (Tymothy grass) E	1.43
Pla a (London plane tree) E	1.78
Pru ar (apricot) E	1.54
Pru du (sweet almond) E	2.62
Pru p (peach) E	1.30
Rat n (common rat) E	7.96
Rat n 4 (common rat) M	1.67
Sola l (tomato) E	4.84
Sola m (eggplant) E	3.67
Tri a (bread wheat) E	3.67
Tri tp (Polish wheat) E	10.00
Zea m (maize) E	9.11

Reference values: negative test result < 0.01 FIU/mL, inconclusive test result 0.01–0.30 FIU/mL, positive test result > 0.30 FIU/mL

*FIU* focus-inducing units

Due to the presence of upper gastrointestinal (GI) symptoms (heartburn, acid regurgitation, foul taste in the mouth), the patient was referred to the Gastroenterology Department at Medical University of Warsaw to undergo diagnostic assessments for eosinophilic esophagitis. At the Gastroenterology Department the patient underwent gastroscopy with esophageal and gastric biopsy. Neither the gastroscopy nor microscopic examination of the biopsy samples revealed any upper GI tract abnormalities. Eosinophilic esophagitis was excluded. Since Helicobacter pylorii was detected, appropriate treatment was administered (500 mg metronidazole 3 times a day, 500 mg tetracycline 4 times a day, 120 mg bismuth oxide 4 times a day, 40 mg pantoprazoleonce a day). Following the course of treatment, the patient’s GI symptoms resolved completely. Currently, the patient remains under observation in an outpatient setting (at our clinic). The patient was recommended to carefully avoid any future contact with teff flour and milk thistle. Additionally, the patient received an emergency kit containing three 10-mg prednisone tablets, three cetirizine tablets, and a pre-filled syringe with adrenalin (EpiPen Senior). Moreover, the patient received thorough training on how and when to use the drugs from his emergency kit. Due to the patient’s diagnosis of wasp venom allergy (based on his medical history and serum specific IgE test results), he was also qualified to undergo venom immunotherapy (VIT), with the treatment scheduled to begin in September 2019.

## Discussion

This paper presents an exceptional case of a patient’s allergy to milk thistle and teff grass. The allergy to milk thistle developed most likely due to exposure at work, while packaging powdered plant matter at a production facility. We would like to emphasize that the patient had never ingested milk thistle in the form of tablets, infusions, teas, seeds, or food additives. We suspect that his Abyssinian love grass (teff-flour) allergy developed via the gastrointestinal route during the sampling of flour and flakes made of the plant. The patient had had no contact with these plants earlier in his past. The available relevant literature contains no reports on teff allergies and only one case report describing a rash that developed following ingestion of milk-thistle tea. Our patient underwent SPT, serum specific IgE test, Faber test (with allergen molecules), and skin tests with native alimentary allergens brought by the patient. The diagnosis was based on the results of these tests and the patient’s medical history. We refrained from conducting any allergen provocation tests due to his past history positive for anaphylactic reaction. We would like to emphasize that there are no commercially available test kits containing the allergens in question. It is to be emphasized that in this particular case the diagnosis was based on suggestive medical history and on positive results of test with native allergens (prick by prick), conducted with the use og products samples provided by the patient. Choice of a diagnostic method (tests with native allergens) proving the correlation with clinical symptoms was driven by lack of available commercial test kits containing allergens of teff and milk thistle. The results of native test were also positive for rice, corn, barley and oat flour, but the patient didn’t present symptoms of food or inhalant allergy to this allergen. On the other hand, skin prick tests were positive for many food and inhalant allergens, including hen’s egg, hazel nuts, rye and wheat flour, banana, anise, pollen of alder, hazel and birch and grass pollen, but with no relation to clinical symptoms. Likewise, sIgE tests, positive for oak, alder, grass, buckwheat, peanuts, sesame, rice and wheat flour did not relate to clinical symptoms. Undoubtedly, multiplex positive tests are a prove of atypical phenotype of our patient. But only one, clinically suspected allergy proven by positive test results was wasp allergy. Similarly, results of Faber test has shown positive results for many allergens, but were not supported by clinical picture. Based on these results, we dare to conclude that the patient presents isolated allergy to teff and milk thistle. Since both teff and milk thistle allergens are of plant origin, cross-allergy between these two is theoretically possible. Nevertheless, these two species belong to different plant families—teff is a cereal plant of the Poaceae family, while milk thistle is an annual plant of Asteraceae. Unfortunately, since allergens extracts of teff and milk thistle are not available we are not able to perform in vitro tests. We hope that, due to widespread use of teff and milk thistle, production of these allergens’ extracts will be possible in the future. We also agree that the results (positive histamine and negative control test) rule out dermographism, that could, in some ways, bias the results of testing.

Both teff and milk thistle have been presented in the literature (both domestic and foreign) in a positive light, the former as a new part of gluten-free diet, and the latter as a treatment for a number of conditions, particularly those of the liver. Gluten-free diet is a treatment of choice in patients with celiac disease and both IgE-mediated and non-IgE-mediated gluten allergies. Moreover, recent years saw attempts to use this diet in prevention and treatment of autoimmune and allergic conditions. Maintaining a gluten-free diet requires a sustained, conscious effort to pick certain foods and refrain from consuming other, main stream foods, which may adversely affect the patients’ quality of life. Moreover, a number of gluten-free products have unremarkable taste and relatively poor nutritional value, hence there is a constant search for new well-tolerated cereals [1, 10]. Teff is a gluten-free cereal derived from Africa and characterized by an exceptional nutritional value. Teff contains proteins (8.7–11.1%), including glutelins, prolamins, albumins, and globulins. Another important component of teff is starch, whose granules form multiform conglomerates. Starch contains 20–26% of amylose. When used in baked foods, starch assists with texture and moisture retention. Fat comprises approximately 2.6–3.8% of teff grain mass [10–12]. A number of domestic and foreign publications have emphasized the therapeutic effects of milk thistle components in many diseases. Moreover, many studies showed hapato protective effects of silymarin via free-radical reduction, increased glutathione synthesis, enhanced hepatocyte regeneration through stabilizing their cellular membranes [5]. A study by Zhong et al. [9] demonstrated beneficial effects of silymarin in patients with nonalcoholic fatty liver disease. The study showed that silymarin administration significantly reduced aminotransferase (AST and ALT) levels in such patients. Umetsu et al. [13] showed silybin to inhibit hepatitis virus cell entry. Moreover, a treatment with silybin in combination with entecavir significantly reduced HBV DNA levels within hepatocytes. There are a number of publications emphasizing beneficial effects of silymarin in metabolic diseases [13, 14]. For instance, in diabetic patients, silymarin lowers triglyceride levels, improves insulin resistance, and exerts a cytoprotective effect on pancreatic beta cells [14, 15]. Furthermore, for a number of years silymar in and/or silybin have been known as carcinogenesis inhibitors. This anticancer effect is mainly due to inducing apoptosis in various neoplastic cells. Certain components of milk thistle are believed to be potentially effective as an adjunct to chemotherapy in cancer patients [16, 17].

## Conclusion

This case report presents the otherwise beneficial teff as a new allergen. Due to the growing tendency to use “new”, previously unknown in Europe, species of cereals as part of gluten-free diet, there might be more cases of new allergies. Practically all existing scientific articles present milk thistle as a substance of substantial diversified medicinal properties. Notably, the presented case report shows this plant from a completely new perspective—as a new allergen. The plant’s increasing popularity might result in an increasing incidence of milk thistle allergy.

## Data Availability

All data generated or analysed during this study are included in this published article.
